# Zinc and Sepsis

**DOI:** 10.3390/nu10080976

**Published:** 2018-07-27

**Authors:** Wiebke Alker, Hajo Haase

**Affiliations:** 1 Department of Food Chemistry and Toxicology, Berlin Institute of Technology, 13355 Berlin, Germany; alker@tu-berlin.de; 2 TraceAge—DFG Research Unit on Interactions of Essential Trace Elements in Healthy and Diseased Elderly, Potsdam-Berlin-Jena, Germany

**Keywords:** zinc, sepsis, biomarker, supplementation, homeostasis

## Abstract

Sepsis, defined as a “life-threatening organ dysfunction caused by a dysregulated host-response to infection” is a major health issue worldwide and still lacks a fully elucidated pathobiology and uniform diagnostic tests. The trace element zinc is known to be crucial to ensure an appropriate immune response. During sepsis a redistribution of zinc from serum into the liver has been observed and several studies imply a correlation between zinc and sepsis outcome. Therefore the alterations of zinc concentrations in different tissues might serve as one part of the host’s defense mechanism against pathogens during sepsis by diverse mechanisms. It has been suggested that zinc is involved in nutritional immunity, acts as a hepatoprotective agent, or a differentiation signal for innate immune cells, or supports the synthesis of acute phase proteins. Further knowledge about these events could help in the evaluation of how zinc could be optimally applied to improve treatment of septic patients. Moreover, the changes in zinc homeostasis are substantial and correlate with the severity of the disease, suggesting that zinc might also be useful as a diagnostic marker for evaluating the severity and predicting the outcome of sepsis.

## 1. Introduction

Zinc is of fundamental importance for the immune system and is involved in different pathologies. In recent years, indications have appeared that zinc homeostasis might be an important factor during sepsis. The following review focuses on the alterations of zinc homeostasis during sepsis and possible physiological functions of this process. It further discusses potential risks and benefits of zinc supplementation as well as a possible approach for using serum zinc as a biomarker for sepsis. 

### 1.1. Sepsis

The term “sepsis” in relation to a disease has already been used by Hippocrates, but to this day it remains a challenge to compile a definition comprising its complexity [1]. This results from the fact that sepsis is rather a syndrome than an illness, showing a not yet fully elucidated pathobiology, and with uniform diagnostic tests still lacking [2]. Sepsis is responsible for about 6 million deaths per year, making it a critical illness and one of the major causes of mortality worldwide [3,4]. Its epidemiological burden is assumed to be much higher in low- and middle-income countries and the mortality rate is affected by the global national income [3,5]. 

There is an urgent need for an easily understandable definition in order to establish public awareness, as well as for improved and uniform diagnostic guidelines for an early recognition of sepsis [2,3]. In the past, different task forces have approached these issues [6,7]. A recent consensus defined sepsis as a “life-threatening organ dysfunction caused by a dysregulated host-response to infection” (Sepsis-3) [2]. To diagnose organ dysfunction in the clinical setting, Singer et al. recommend the Sequential Organ Failure Assessment (SOFA) score. It includes parameters to evaluate the functions of respiration, the liver, the cardiovascular system, the central nervous system, the kidneys, and coagulation. An elevation of the total SOFA score of 2 points or more indicates organ dysfunction [2,8].

Sepsis is initiated by an infection [2]. The pathogen triggers an immune response, comprising pro-inflammatory mechanisms to defeat the pathogen and regenerate the affected tissue, as well as subsequent anti-inflammatory mechanisms to counteract the pro-inflammatory actions in order to limit collateral damage in healthy tissue [9,10]. A dysregulation of this immune response, as it appears during sepsis, leads to an over-reaction of the immune system, which can affect both mechanisms described. Hyper-inflammation in the form of a systemic inflammatory response syndrome (SIRS) can lead to a damage of the host’s own tissue. Immune-suppression, also known as compensatory anti-inflammatory response syndrome (CARS), leaves the host more vulnerable to secondary infections [2,11,12]. A wealth of literature is provided about sepsis and its symptoms, diagnostics, and possible medical treatment approaches (e.g., [10,11,13,14]), to which the reader is referred for more detailed information on these aspects of sepsis.

### 1.2. Zinc

Zinc is an essential trace element [15,16]. In the body it functions, for example, as a co-factor for a high number of enzymes or as a structural element for a variety of proteins [17]. Zinc deficiency can result in growth retardation, dermatitis, and hypogonadism, or symptoms such as delayed wound healing, thymic atrophy or lymphopenia, and high incidence of infection; the latter points are due to its particular importance for the immune system [18,19,20]. Consequently, zinc deficiency results in multiple immunological changes, including what seems to be a shift toward a predominantly innate immune response when the availability of zinc is limited [21]. One particularly important effect of zinc is a modulation of the production of inflammatory cytokines [22]. Moreover, zinc is crucial for the functioning of virtually all immune cells. For example, the differentiation of immature T-cells depends on zinc, because thymulin, a hormone involved in T-cell differentiation, depends on zinc as a co-factor [23,24]. In addition, the maturation of T-cells is influenced by their zinc status. On the one hand a deficiency results in altered ratios of Th1- and Th2-cells, an increased apoptosis-rate of immature T-cells, and consequently a decrease in T-cells in total [21,25,26,27]. On the other hand, zinc supplementation has also been shown to promote regulatory T-cell development and to suppress the maturation of Th17-cells, therefore having an inhibitory effect on Th17-mediated autoimmune-diseases [28,29,30].

On the molecular level, some functions of zinc have been linked to its role as a second messenger in immune cells. It has been shown that alterations in the intracellular free zinc-concentration function as a “zinc signal”. Such a change in the intracellular free zinc concentration is induced by the binding of various ligands to their respective receptors, such as lipopolysaccharide (LPS) to Toll-like receptor 4 (TLR-4), or the corresponding antigens to immunoglobulin E when it is present on the high-affinity immunoglobulin E-receptor (FcεRI). Different kinds of immune cells vary in their expression of receptors that utilize zinc; consequently zinc signals mediate diverse events, for example, formation of pro-inflammatory cytokines by monocytes [31], presentation of major histocompatibility complex (MHC) class II molecules at the surface of dendritic cells [32], formation of neutrophil extracellular traps by neutrophil granulocytes [33], or proliferation of T-cells [34].

The essentiality of zinc for the immune system has been known since the 1960s and the corresponding mechanistic knowledge has been expanding ever since. Its importance for the immune system is based on various different mechanisms, each in its own way essential to ensure the functionality of the immune system and the accurate processes of immune response, especially for inflammatory processes. As a complete summary would exceed the scope of this article, the reader is referred to recent review articles on the subject of zinc and immunity for more comprehensive information [35,36] as well as to a recent review on the protective role of zinc during sepsis [37].

## 2. Zinc Homeostasis during Sepsis

Zinc has not only a crucial role in ensuring a proper immune response. Another observation in the context of zinc and the immune system is an altered zinc homeostasis of the host during an infection, which is discussed below.

### 2.1. Changes in Zinc Homeostasis

The host’s response to an infection or injury is referred to as an acute phase reaction (APR). This process aims to defeat the insult, take actions against ongoing tissue damage, and re-establish homeostasis. One of the characteristics of APR is hypozincemia. To study the time course of hypozincemia and examine possible underlying mechanisms, Gaetke et al. injected LPS to healthy volunteers in order to induce an inflammatory response. Subsequently, an increase in serum tumor necrosis factor α (TNF-α) and interleukin-6 (IL-6) was observed, followed by a decrease in serum zinc concentrations. To explain hypozincemia in their model of infection the authors suggested an internal redistribution of zinc, mediated by cytokines [38]. The analysis of serum from sepsis patients in the intensive care unit (ICU) revealed that serum zinc concentrations were reduced compared to a healthy control group or the normal physiological range [39,40,41]. Probably these differences were not caused by a zinc-deficient state due to malnutrition, but redistribution of zinc within the patients’ bodies. Consistently, a study by Hoeger et al. showed a time-dependent decline of the serum zinc concentrations after induction of sepsis in a porcine model [42]. In order to reveal the mechanisms responsible for the observed hypozincemia, Luizzi et al. used a mouse model and induced inflammation either by turpentine or LPS. Zrt-, Irt-like protein (ZIP)14 mRNA was the transporter transcript that was upregulated the most. This upregulation was liver-specific and an increase of ZIP14 on the plasma membrane of hepatocytes was shown. Further studies indicated a role of the inflammatory cytokines IL-6 and IL-1β in the upregulation of ZIP14. Also, an increase in metallothionein (MT)-1 mRNA in the liver has been observed [43,44]. This observation of enhanced MT expression has been described before in the context of APR [45,46,47]. As reviewed in detail before, MTs function as intracellular metal-binding proteins and are crucial to maintain the intracellular zinc homeostasis. Their expression is induced by a number of metals, one of them zinc [48]. The increased liver zinc concentrations accompanying hypozincemia lead to an enhanced need for zinc-binding proteins in order to ensure the intracellular zinc homeostasis. The production of MT in the liver seems to be regulated by cytokines as well as zinc [49]. Using a murine model, the already-described decline in serum zinc concentration and an increase in liver zinc level were observed after induction of sepsis. The analysis of the time-course of mRNA expression in the liver first showed a successive upregulation of ZIPs 4, 6, and 10 within the first day, which then returned to near normal levels at 72 h after induction of sepsis. The mRNA expression of ZIP14 increased at 9 h and stayed upregulated for the time of the investigation (72 h), which is in line with the observations described previously and supports a major role for ZIP14 in the redistribution of zinc during sepsis [50]. Taken together, the APR comprises a fundamental change in liver zinc homeostasis and apparent zinc deficiency in the serum [50,51].

The studies mentioned so far aimed, among other things, on understanding the mechanisms responsible for the observed hypozincemia and to track the redistribution of zinc in the body. An alternative approach to broaden the understanding of how certain processes are changed during sepsis is gene expression analysis. The method allows insights into the impact of sepsis at the translational level. The analysis of blood samples from pediatric septic shock patients showed a regulation of genes that are involved in a large number of signaling pathways and gene networks, especially those related to immunity and inflammation. Also, it has been shown that up to 12% of the gene probes that showed a significantly decreased expression compared to the control group are associated with the categories of “zinc, zinc finger, metal-binding and zinc-ion binding” [52]. These results suggest a repression of genes involved in zinc homeostasis, or depending on an intact zinc homeostasis, as a significant feature of pediatric septic shock [52,53,54,55]. Further, the question arises as to whether differences in gene expression in pediatric septic shock survivors and non-survivors can be observed. Regarding zinc homeostasis, two isoforms of MT have been identified that showed an increased expression in non-survivors compared to survivors. In addition, non-survivors had a significantly lower serum zinc concentration compared to survivors. Considering the zinc-binding properties of MT, Wong and colleagues interpret these results to indirectly imply that increased MT expression in non survivors might affect zinc homeostasis and thereby serum zinc concentration [53].

Taken together, different approaches imply a contribution of zinc and its altered homeostasis to the pathobiology of sepsis. 

### 2.2. Possible Reasons for the Redistribution of Zinc

**Figure 1:**
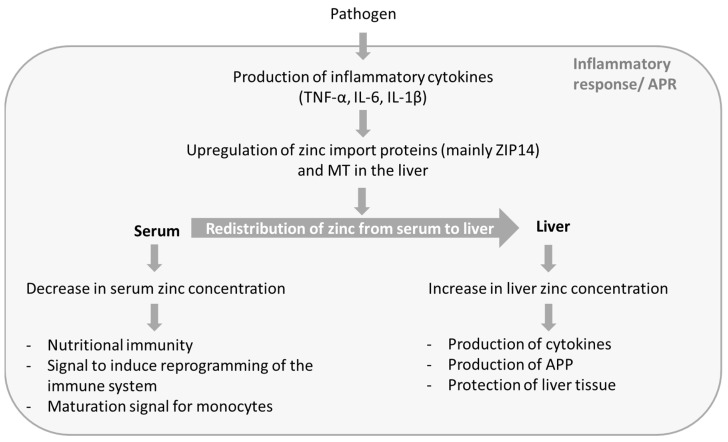
Possible functions of zinc in sepsis. During the APR of sepsis zinc is redistributed from serum to liver. This process results in decreased serum zinc concentration and increased liver zinc. The altered zinc concentrations seem to serve different functions and to be a part of the host’s defense against pathogens. APR: acute phase reaction; IL: interleukin; TNF: tumor necrosis factor; MT: metallothionein; APP: acute phase proteins.

With respect to the considerable differences of serum zinc concentrations between sepsis patients and the corresponding control groups, as well as the finely tuned alterations of zinc homeostasis, it can be assumed that these are part of a directed process that aims to benefit the host in defeating the pathogen. This process includes a decrease in serum zinc concentration as well as an increase in liver zinc concentration, whereas both aspects seem to benefit the host’s defense against pathogens. Figure 1 gives a brief overview of the processes causing the alterations in zinc concentrations, as well as the possible beneficial effects. 

Research on zinc homeostasis in the context of sepsis delivered a variety of explanations for the beneficial effects of a redistribution of zinc. The respective studies are discussed below.

One of the main effects of the redistribution of zinc is an accumulation of zinc in the liver. Hence, it seems as is if a higher liver zinc level might benefit the host during infection. Among other things, the APR is not only characterized by the previously mentioned redistribution of zinc, but also by production of acute phase proteins (APP) and the release of cytokines [56,57]. Zinc serves as an important structural element for many proteins and is required by enzymes involved in transcription and translation. Therefore, the higher synthesis rate of APP in the liver could cause an increased requirement for zinc during APR [56,57,58]. With respect to cytokine production a knockout (k.o.) of ZIP14 in mice, a transporter important for the regulation of zinc homeostasis in hepatocytes, showed lower mRNA expression of TNF-α, IL-6, IL-1β, and IL-10 in the liver compared to wild-type (w.t.) mice after induction of sepsis in a murine model. Simultaneously, plasma levels of TNF-α, IL-6, and IL-10 were significantly higher in k.o. than in w.t. mice. The results indicate a disadvantage of the ZIP14 k.o. mice during sepsis based on increased markers of inflammation and an influence of zinc, transported by ZIP14, on the production of cytokines during APR. However this observation is surprising, because a decrease in mRNA expression would be expected to result in lower cytokine levels. Possible explanations would be elevated cytokine expression elsewhere in the body, or an impact on mechanisms mediating the expression of antagonists of the pro-inflammatory cytokines [50].

Other studies suggest that the altered zinc supply during endotoxemia has a major influence on energy production in the liver. Injection of LPS caused an increase in hepatic zinc and MT in w.t. mice, whereas zinc levels stayed unchanged in MT k.o. mice. At the same time the liver glucose of the former stayed unchanged while the levels in MT k.o. mice decreased significantly. These results imply a lack of hepatic gluconeogenesis in the MT KO mice and a role for MT, and most likely also for zinc, in maintaining glycaemia after induction of an infection [59]. 

A protective role of zinc for the liver has also been suggested. Using murine models it was shown that after injection of endotoxin, zinc-deficient nutrition resulted in enhanced lipid peroxidation in the liver compared to the zinc adequate group [60]. Another study showed that zinc pre-treatment of mice resulted in an increased intracellular availability of zinc in liver cells and was accompanied by decreased accumulation of superoxide and necrotic cell death in the liver after injection of LPS [61]. Both experimental observations support a protective role of zinc in the liver during infection. Interestingly, after injection of endotoxin, Sakagouchi et al. saw an increase in MT only in the zinc adequate group, but not in zinc-deficient animals, and therefore suggested a relation between zinc concentration, endotoxin-induced MT and lipid peroxidation [60]. In contrast, Zhou et al. observed the protective effects of zinc pre-treatment on liver cells in w.t. mice as well as MT k.o. mice, leading them to propose an effect of zinc independent of MT [61]. These results are not necessarily contradictory, but could imply that the protective effect of zinc on the liver could work in more than one way. Further studies on this topic would be useful, since organ dysfunction is one of the hallmarks of sepsis and a better understanding of its mechanisms is the basis of a possible prevention. In summary, studies show multiple and diverse functions of zinc in the liver during the onset of sepsis, suggesting a physiological basis for the accumulation of zinc.

The redistribution of zinc and accumulation in the liver is accompanied by a decrease in serum zinc concentration. With regard to the host’s defense against pathogens, this effect might have some benefits as well. One protective mechanism of the host is referred to as nutritional immunity. Pathogens, just like all living organisms, require transition metals for their survival. The host’s strategy is to restrict the pathogens’ access to essential transition metals, for example by lowering their concentrations in the serum or secretion of metal ion binding proteins. This process is not limited to zinc but has been described for other micronutrients, such as iron or manganese [62].

A decrease in the serum zinc concentration has also been shown to influence the respective number and maturation of immune cells. Therefore, the alteration of serum zinc during the APR might function as a signal. Using a murine model of zinc deficiency, a downregulation of lymphopoiesis and upregulation of myelopoiesis was found [19,21,27,63]. In line with this observation, a decrease of intracellular zinc occurred as a result of homeostatic changes during monocytic differentiation of HL60 cells. Moreover, experiments simulating zinc deficiency showed that lower zinc levels promoted the development of HL-60 cells along the myeloid lineage into functionally mature macrophages [64].

The immune cells that benefit from a decline of serum zinc are part of the innate immune system. They represent the first line of host defense and provide a faster response than the cells of the adaptive immune system. Fraker and King proposed the hypothesis of a “reprogramming of the immune system” during zinc deficiency in the form of a shift from adaptive immunity to predominantly innate immunity [65]. Here, limited resources would be directed toward an immediate defense on the expense of long-term protection. In the context of sepsis, the reduction of serum zinc as a promoting signal for the innate immune system might be an attempt to focus the defense mechanisms toward a fast innate immune reaction in the face of a potentially overwhelming infection.

The various aspects by which zinc homeostasis seems to be involved in the body’s defense against pathogens suggest that the redistribution of zinc in the course of sepsis could serve multiple physiological purposes. However, further research will be required to evaluate the physiological significance of the different processes, thereby widening our understanding of the zinc-dependent endogenous defense mechanisms. This knowledge is required to develop medical approaches in order to support the host’s body and its defense during sepsis.

### 2.3. Possible Adverse Effects of Zinc-Redistribution

Despite the potential physiological roles of the redistribution of zinc, this process might also cause some adverse effects, especially the decrease in serum zinc concentration. Possible symptoms of a low serum zinc concentration due to zinc deficiency caused by malnutrition are higher levels of pro-inflammatory cytokines, higher markers of oxidative stress, and oxidative damage to proteins, lipids, and DNA [66,67,68,69,70]. Notably, zinc deficiency and sepsis show several parallels in addition to low serum zinc concentrations. The severe effects of zinc deficiency on the functionality of the immune system have already been described in the introduction, including a decrease in T-cell numbers and function [20]. Dysfunction and apoptosis of T-cells have also been observed in the context of sepsis [71,72,73]. Other symptoms of zinc deficiency have been found during sepsis as well, for example the overproduction of pro-inflammatory cytokines and SIRS, which is a hallmark of sepsis and a major factor in the host’s dysregulation to an infection during sepsis [11]. Furthermore, effects such as lipid peroxidation and oxidative damage of DNA, proteins, and mitochondria have been observed [74,75,76]. In light of the parallels between symptoms of zinc deficiency and sepsis, further research should elucidate the question whether the body’s reaction to an infection might cause a decrease in bioavailable zinc severe enough to contribute to, or maybe even cause, these common symptoms during sepsis.

In this context it is of interest to mention that several studies have shown a correlation between a patient’s serum zinc concentration and the severity of the inflammatory response or sepsis. In critically ill patients, those with a high SOFA score showed a significantly lower serum zinc concentration than patients with a low SOFA score, whereby a higher SOFA score was associated with higher mortality [2,58]. In line with these results serum zinc concentrations were found to be inversely correlated with the SOFA score in other studies, as well [39,77]. Yet another study revealed a significantly lower serum zinc concentration in sepsis patients who developed a recurrent sepsis compared to those that did not. Additionally, sepsis non-survivors had a significantly lower serum zinc concentration than survivors [41]. Because zinc fulfils a great number of crucial functions in the body, and especially the immune system, it seems reasonable that a decrease of bioavailable zinc in the serum could contribute to some adverse effects, thereby aggravating sepsis. 

## 3. Zinc Supplementation

The correlation between low serum zinc concentrations and a higher mortality rate or chance of recurrence raises the question, if supplementation of zinc might be a treatment option to improve the outcomes of sepsis. Table 1 gives an overview of zinc supplementation studies in the context of sepsis in humans. In these studies zinc supplementation took place after the onset of sepsis. Some of them show a beneficial effect of zinc in form of a lower mortality rate and a better neurological development of neonates [78,79,80]. However, it was also observed that supplementation did not result in any significant differences between the zinc group and the control group [81] or even showed a harmful effect [82]. The published animal studies mostly examined the effects of prophylactic zinc supplementation (Table 2). In several of them the supplementation of zinc prior to induction of sepsis showed beneficial effects, such as improved survival, lower serum concentrations of pro-inflammatory cytokines, lower bacterial burden or improved pulmonary function compared to the control-group [50,83,84,85]. However, for the animal studies the results are also not consistent and a missing effect of zinc supplementation is reported [86] as well as a harmful outcome in a case where zinc was applied during the acute phase [87]. The negative results of zinc supplementation may be explained by the fact that the reduction of zinc levels during sepsis may occur for a reason, such as the above-mentioned physiological functions. High doses of zinc were shown to have a pro-inflammatory effect [88] and might therefore aggravate inflammation, or zinc supplementation could potentially interfere with nutritional immunity, one of the endogenous defense mechanisms based on a shortage of zinc in the serum [62].

Another important factor is the bioavailability of serum zinc. Albumin is the major zinc-buffering protein [89] as well as a negative APP [56,90]. Its concentration decreases during sepsis, leading to a decreased zinc-binding capacity of the serum [42]. As a consequence, supplementing sepsis patients until the “normal” total serum zinc concentration is restored would result in a supraphysiological concentration of free, and thereby bioavailable, zinc in these patients.

Correcting zinc deficiency prior to sepsis is certainly beneficial, but difficult to realize, as in most cases sepsis cannot be predicted. Moderate supplementation during sepsis might, in some cases, turn out to be helpful, as well. This may be particularly so in patients with pre-existing zinc deficiency that is so pronounced that the liver cannot accumulate sufficient amounts to exert the abovementioned protective effects of zinc. However, as the reduction of serum zinc seems to be a necessary physiological process, in these cases extreme care needs to be taken in order not to exceed the zinc-binding capacity of the serum to avoid negative effects, such as counteracting nutritional immunity or aggravating inflammation. 

The endogenous processes, which are supposed to be affected by the supplementation of zinc, are quite complex and fine-tuned. As illustrated by the divergence of the study results, effective zinc supplementation has to be just as elaborate with regard to timing as well as dosage in order to achieve optimal results. 

## 4. Serum Zinc Concentration as a Possible Biomarker for Sepsis

In addition to a potential therapeutic value of zinc supplementation, the altered zinc homeostasis could have potential to be utilized for establishing a biomarker based on the serum zinc level of a patient. In a porcine model of sepsis, the decline of the serum zinc concentration was the first marker of inflammation, already statistically significant one hour after induction of sepsis. Thereby, it was an earlier indicator for inflammation than the increase of the pro-inflammatory cytokines IL-6 and TNF-α, which reached statistical significance two hours after induction of sepsis [42]. However, hypozincemia is activated in non-infectious as well as infectious inflammation. The ability to discriminate between the different causes for hypozincemia would be relevant for a use of zinc as a biomarker for sepsis. So far the studies vary in their results as to whether there is a significant difference in the serum zinc concentration of sepsis patients and a critically ill control group, surgical control group, or trauma patients [39,41,91]. Further studies would be required to evaluate the potential of serum zinc concentration as a biomarker for sepsis, including large clinical studies and the evaluation of clinical data sets to proof its validity and prospective value.

According to Singer et al., a diagnostic marker for sepsis should be easily to obtain and available promptly at reasonable cost [2]. Provided the evaluation studies on serum zinc concentration as a biomarker for sepsis turn out to be positive, this marker would fulfil Singer et al.’s demands [2]: It would allow for early recognition, which is crucial to improve the patient’s outcome and decrease the sepsis-related mortality rate. Also, patient serum is easily to obtain and the parameter could be monitored closely once the patient is under medical supervision. If the required infrastructure is available, an analysis of the serum zinc concentration delivers fast results at reasonable cost. 

One hindrance for the use of zinc as a marker in sepsis might be the necessary infrastructure. Presently, atomic absorption spectrometry or inductively-coupled plasma mass spectrometry are being used to quantify zinc. Even with the required instruments available the quality of the results strongly depends on the analytical abilities of the performing personnel or laboratory [92]. If this sophisticated equipment is not available, e.g., in rural areas or countries with a less developed infrastructure, sending the samples to external labs might cause significant delay in obtaining these time-sensitive results, nullifying the advantage of an early diagnostic marker. Some basic work on easier point-of-care devices to measure the serum zinc concentrations has been reported, but these still have to reach clinical applications [93,94].

Other tools widely used to detect zinc in biological samples are fluorescent probes [95]. However, they have only sparsely been used in the context of sepsis thus far. One example is the application of Zinpyr-1 by Hoeger et al. in serum samples from a porcine model of sepsis [42]. It should be noted that these probes do not measure total zinc, but determine the amount of free zinc in the sample. Still, this parameter might be interesting to look at in the context of sepsis, because it represents the amount of bioavailable zinc and has been suggested as an alternative biomarker for a person’s zinc status [96]. 

Some aspects regarding the mechanism mediating the redistribution of zinc have not been fully revealed yet. Once understood, they might contribute to an even better use of zinc homeostasis as a diagnostic parameter in sepsis. Hoeger et al. observed a continuous decline of serum zinc concentration from one hour after induction of sepsis in vivo. However, for up to two hours no induction of mRNA expression of MT-1, MT-2, and ZIP14 was observed in an in vitro model of hepatoma cells after incubation with IL-1β, IL-6, or LPS [42]. Wessels et al. reported the lowest serum zinc concentration in their sepsis model at 9 h after induction whereas the upregulation of ZIP14 in the liver and the increase in liver zinc have been observed from hour 9 on after induction [50]. These studies raise further questions, mainly as to how the decline of serum zinc concentration is mediated in the initial phase, and if zinc may be located in some other part of the body in the period from its disappearance from the serum until it is finally detected in the liver.

## 5. Conclusions

Zinc is an essential trace element and has been shown to be crucial for ensuring an adequate immune response. In the context of sepsis the host’s zinc homeostasis is altered. Various study results imply some of the alterations to be part of the host’s defense mechanism against pathogens (Figure 1). There are indications that a patient’s zinc supply and serum zinc concentration is associated with severity, outcome, and recurrence of sepsis. Zinc seems to have potential to be used as a biomarker or even as a starting point for a therapeutic approach. Further research is required to broaden the understanding of zinc homeostasis during sepsis and the underlying mechanisms as well as to evaluate the possible clinical applicability of this knowledge. 

## Figures and Tables

**Table 1:** Zinc supplementation studies in septic patients.

Study Population	Intervention/Zn-Supply	Observation Time Points	Results (Zinc Group vs. Control Group)	Reference
Neonates with clinical signs suggestive of sepsis and at least two screening tests positive	Zinc group *: Antibiotic treatment, dose of 3 mg/kg bodyweight (BW) zinc sulfate monohydrate twice a day for 10 days (corresponding to 2.1 mg/kg BW Zn^2+^ per day) Control group: Antibiotic treatment	Measurement of blood samples from base line (BL) and after 10 days	• Significant increase in serum zinc concentrations compared to BL • Significant decrease in TNF-α compared to BL • Lower mortality rate, but not reaching significance (7.4% compared to 16.4%) • Similar duration of hospitalization	[78]
Neonates with clinical features of sepsis and positive blood culture or positive sepsis screening tests	Zinc group *: Antibiotic treatment, dose of 3 mg/kg BW zinc sulfate monohydrate twice a day for 10 days (corresponding to 2.1 mg/kg BW Zn^2+^ per day) Control group: Antibiotic treatment	Measurement of blood samples from BL and after 10 days	• Increase in serum zinc concentrations compared to BL, but not reaching significance • Lower mortality rate, but not reaching significance (4.5% compared to 13.6%) • Better neurological status (chance of having abnormalities is 70% less) at one month of age • Similar duration of hospitalization	[79]
Neonates with clinical manifestations of sepsis who exhibited two positive screening tests	Zinc group *: Antibiotic treatment, dose of 3 mg/kg BW zinc sulfate monohydrate twice a day for 10 days (corresponding to 2.1 mg/kg BW Zn^2+^ per day) Control group: Antibiotic treatment	Measurement of blood samples from BL and after 10 days	• Significant increase in serum zinc concentrations • Significantly lower mortality rate (6.6% compared to 17.3%) • Better neurodevelopment (significantly better Mental Development Quotient) at 12 month of age	[80]
Neonates with probable sepsis	Zinc group *: Antibiotic treatment, dose of 1 mg/kg BW zinc sulfate per day until the final outcome (discharge/death) (corresponding to 0.4 mg/kg BW Zn^2+^ per day) Control group Antibiotic treatment, dose of placebo until the final outcome (discharge/death)	Final outcome at discharge/death	• No significant differences in mortality rate • No significant differences in duration of hospital stay • No significant differences in requirement of antibiotic treatment	[81]
Patients with pancreatitis or catheter sepsis	Zinc group *: Total parental nutrition, 30 mg zinc sulfate per day for 3 days (corresponding to 12.1 mg Zn^2+^ per day) Control group: Total parental nutrition, 0 mg zinc sulfate for 3 days	Measurement of blood samples from BL, day 1, 2, 3; highest temperatures from patients’ bedside charts from day 1, 2, 3	• Higher temperatures, reaching significance on day 3 • No difference in serum IL-6 and ceruloplasmin	[82]

* In all studies, zinc supplementation was started after the onset of sepsis.

**Table 2:** Zinc supplementation studies in animal models of sepsis. LPS: lipopolysaccharide.

Animals	Sepsis Model	Intervention/Zn-Supply	Results (Zinc Group vs. Control Group)	Reference
Male mice (C57BL/6)	Intraperitoneal (i. p.) fecal slurry injection; sacrifice of mice at 24 h to conduct assays or observed 72 h for survival study	Zinc group: Injection of 10 mg/kg BW zinc gluconate every 24 h for 3 days prior to induction of sepsis, injection continued every 24 h after induction of peritonitis (corresponding to 1.4 mg/kg BW Zn^2+^ per day) Control group: Injection of equal volume of saline at the same time points as for the zinc group	• Significantly improved survival following sepsis at 72 h after induction • Significantly lower myeloperoxidase activity in lung tissue (at 24 h) • Significantly lower bacterial burden in blood and spleen (at 24 h) • Significantly lower serum keratinocyte chemoattractant concentration (at 24 h) • No significant difference between serum concentration of IL6, IL-1β, IL-10	[84]
Male and female mice (C57BL/6)	Cecal ligation and puncture; sacrifice of mice at 24 h	Zinc group: High-zinc diet (180 mg/kg) for 7 days prior to induction of sepsis Control group: Zinc-adequate diet (30 mg/kg) for 7 days prior to induction of sepsis	• Significantly lower IL-6 mRNA expression in hepatocytes • Significantly lower TNF-α mRNA expression in hepatic leukocytes • Significantly lower S100A9 mRNA expression in white blood cells • Significantly lower serum concentrations of TNF-α, S100A8 and S100A9 • Significantly lower serum concentration of plasma alanine aminotransferase • Significantly lower bacterial burden in blood and spleen	[50]
Male and female juvenile mice (C57BL/6)	I. p. cecal-slurry injection and measurement of blood samples at 6 h and 12 h; mice were sacrificed at 6 h or 12 h or observed for 72 h for survival study	Zinc group: Injection of 10 mg/kg BW zinc gluconate once a day for 3 days prior to induction of sepsis (corresponding to 1.4 mg/kg BW Zn^2+^ per day) Control group: Injection of equal volume of saline for 3 days prior to induction of sepsis	• Significantly improved survival of following sepsis at 72 h after induction • Significantly lower myeloperoxidase activity in lung tissue (at 12 h) • Significantly lower bacterial burden in peritoneal fluid (at 12 h) • Significantly lower serum concentrations of IL-2 (at 6 h, 12 h), IL-6 (at 6 h, 12 h), IL-1β (at 6 h), and keratinocyte-derived chemokines (at 12 h)	[83]
Female farm pigs (Deutsche Landrasse)	Intravenous infusion of LPS and measurement of the parameters for a duration of 300 min after infusion of LPS; pigs were sacrificed at 500 min of total registration time	Zinc group: Infusion of 25 mg/kg BW zinc-bis-(dl-hydrogenaspartate) 24 h prior to infusion of LPS (corresponding to 5 mg/kg BW Zn^2+^ per day) Control group: Infusion of saline 24 h prior to infusion of LPS	• Increased arterial and venous oxygen pressure (reaching significance at 45 min or 210 min) • Increased arterial and venous oxygen saturation (reaching significance at 210 min) • Stable intrapulmonary shunt (instead of an increase in the control group) • Stable extravascular lung water (EVLW) (instead of an increase in the control group)	[85]
Female farm pigs (Deutsche Landrasse)	Intravenous infusion of LPS and measurement of parameters for a duration of 60 min after infusion of LPS; pigs were sacrificed at 60 min and organs removed for analysis	Zinc group: Infusion of 25 mg/kg BW zinc-bis-(dl-hydrogenaspartate) 2 h prior to infusion of LPS (corresponding to 5 mg/kg BW Zn^2+^ per day) Control group: Infusion of saline 2 h prior to infusion of LPS	• Decrease in arterial and venous oxygen pressure (reaching significance at 30 min) • Decrease in arterial and venous oxygen saturation(reaching significance at 30 min or 15 min) • Increase in intrapulmonary shunt (reaching significance at 30 min) • Increase in EVLW (reaching significance at 45 min) • Increase in mean hemoglobin (reaching significance at 30 min) • Increase in IL-6 and TNF-α plasma concentrations (reaching significance at 0 min or 45 min) • Significant higher weights of lungs, width of alveolar septae and rate of paracentral liver necrosis	[87]
Female farm pigs (Deutsche Landrasse)	Intravenous infusion of LPS and measurement of parameters for a duration of 1020 min, with infusion of zinc from 600 to 720 min; pigs were sacrificed at the end of the study period and a necropsy carried out	Zinc group: Infusion of LPS at 0 h, 5 h and 12 h, infusion of 25 mg/kg BW zinc-bis-(dl-hydrogenaspartate)(corresponding to 5 mg/kg BW Zn^2+^ per day) at 10 h during sepsis Control group: Infusion of LPS at 0 h, 5 h and 12 h, infusion of saline at 10 h during sepsis	• Trend to higher arterial and venous oxygen pressure • Trend to higher arterial and venous oxygen saturation • No significant differences in intrapulmonary shunt • No significant differences in EVLW • Different courses in IL-6 and TNF-α plasma concentrations, at the end almost similar levels	[86]
